# The dark side of social ties: coronavirus 2019-induced fear and intergroup conflicts

**DOI:** 10.1192/j.eurpsy.2022.1335

**Published:** 2022-09-01

**Authors:** S. Tei, J. Fujino

**Affiliations:** 1 Kyoto University, Department Of Psychiatry, kyoto, Japan; 2 Waseda University, Institute Of Applied Brain Sciences, Saitama, Japan; 3 Showa University, Medical Institute Of Developmental Disabilities Research, Tokyo, Japan; 4 Tokyo International University, School Of Human And Social Sciences, Saitama, Japan; 5 Tokyo Medical and Dental University, Department Of Psychiatry And Behavioral Sciences, Graduate School Of Medical And Dental Sciences, Tokyo, Japan

**Keywords:** hostility, social tie, intergroup conflicts, flexibility

## Abstract

**Introduction:**

The relationship between fear and social ties has been frequently discussed in the context of the coronavirus 2019 (COVID-19) pandemic, but investigation of the nature of these experiences is still insufficient. Research suggests that people who respect social ties often experience better mental health outcomes. However, when socially isolated, excluded, or subjected to rumors, they may become more vulnerable to criticism, shame, and fear. Another potential problem of the COVID-19 pandemic is intergroup prejudice and distrust.

**Objectives:**

To examine the development and mitigation of social ties, fears, and biases during the COVID-19 pandemic.

**Methods:**

We applied discourse analysis to relevant literature and their associated references that incorporated textual, social, and cognitive dimensions. The main databases used were PubMed and Web of Science.

**Results:**

Although the importance of social ties was loudly vocalized as lessening loneliness, people also globally described stigma-related fear or intergroup conflicts. The sense of social ties appeared disproportionately amplified in the form of an in-group identity, group pressures, and empathic distress. Some people overstated worries about their COVID-19-positive status being revealed to others and causing distress for them. Furthermore, discrimination and vigilantism were manifested with fear-related stereotyping and hostility.

**Conclusions:**

Our findings support the view that social ties can indeed function as both risk and protective factors. Context-adjusted perspectives and reciprocal dialogs seem crucial to alleviate these negative impacts. The subsequent mitigation of misunderstandings, fear-induced bias, and maladaptive distress appraisal may lead to a more reasonable and flexible recognition of social ties.

**Disclosure:**

No significant relationships.

